# Using Raman Spectroscopy as a Fast Tool to Classify and Analyze Bulgarian Wines—A Feasibility Study

**DOI:** 10.3390/molecules25010170

**Published:** 2019-12-31

**Authors:** Vera Deneva, Ivan Bakardzhiyski, Krasimir Bambalov, Daniela Antonova, Diana Tsobanova, Valentin Bambalov, Daniel Cozzolino, Liudmil Antonov

**Affiliations:** 1 Institute of Organic Chemistry with Centre of Phytochemistry, Bulgarian Academy of Sciences, Acad. G. Bonchev str., bldg. 9, 1113 Sofia, Bulgaria; veradeneva@gmail.com (V.D.); dantonova@orgchm.bas.bg (D.A.); lantonov@orgchm.bas.bg (L.A.); 2 Department of Technology of Wine and Beer, University of Food Technologies Plovdiv, 26 Maritza blvd., 4002 Plovdiv, Bulgaria; ivanbak81@yahoo.com (I.B.); bambalovkg@mail.bg (K.B.); dvch11@abv.bg (D.T.); 3 Department of Viticulture, Agricultural University Plovdiv, 12 Mendeleev blvd., 4000 Plovdiv, Bulgaria; bambalovvg@abv.bg; 4 School of Science, RMIT University, GPO Box 2476, Melbourne, VIC 3001, Australia

**Keywords:** Raman, wine, chemometrics, phenolic compounds, Bulgaria

## Abstract

Raman spectroscopy, being able to provide rich information about the chemical composition of the sample, is gaining an increasing interest in the applications of food. Raman spectroscopy was used to analyze a set of wine samples (red and white) sourced from rarely studied traditional Bulgarian wines. One of the objectives of this study was to attempt the fast classification of Bulgarian wines according to variety and geographic origin. In addition, calibration models between phenolic compounds and Raman spectroscopy were developed using partial least squares (PLS) regression using cross-validation. Good calibration statistics were obtained for total phenolic compounds (by the Folin–Ciocalteu method) and total phenolic compounds and phenolic acids (spectrophotometrically at 280 nm) where the coefficient of determination (R^2^) and the standard error in the cross-validation (SECV) were 0.81 (474.2 mg/dm^3^ gallic acid), 0.87 (526.6 mg/dm^3^ catechin equivalents), and 0.81 (44.8 mg/dm^3^ caffeic equivalents), respectively. This study has demonstrated that Raman spectroscopy can be suitable for measuring phenolic compounds in both red and white wines.

## 1. Introduction

In recent years, great efforts were made in the development of accurate and fast analytical techniques, which require no sample preparation [1,2,3,4,5]. Nowadays special attention is given to the use of vibrational techniques for food and beverages authenticity control due to their rapid, automated, low cost, and non-destructive character [1,2,3,4,5]. In addition, the progress made in the field of chemometric methods increased the versatility and application of vibrational techniques (IR or Raman) in the food and beverage industries [1,2,3,4,5]. Infrared spectroscopy (IR) is extensively used for different purposes in the wine industry worldwide, as reviewed and reported by other authors [3,4,5]. These techniques are well suited for routine analysis as they are easy to use under industrial conditions. Unfortunately, its main disadvantage is the limitation for the water-rich sample assessment (e.g., wine samples), due to the strong absorption bands of water in the IR region [3,4,5]. On the contrary, Raman spectroscopy appears to be more suitable for the vibrational assessment of water-containing samples, due to the relatively weak water signals in the vibrational fingerprint range [6,7].

Nowadays, Raman spectroscopy, that can provide rich information about the chemical composition of the sample, has gained wide interest in the applications of food as scattered light by the organic molecules. The use of Raman spectroscopy has been explored for the analysis of wine samples by several authors where different applications, such as the identification and determination of some wine compounds (e.g., anthocyanidins and total phenolic content) [8,9,10,11], and wine quality control [6,10] are the most significant studies reported in the literature. However, the main analytical advantages of Raman spectroscopy are not fully appreciated or evaluated for the analyses of wine.

Traditional Bulgarian wines have never been intensively studied [12,13,14,15,16,17]. This study provides a great opportunity to attempt fast classification in the direction of variety and geographic origin of wines produced in Bulgaria using Raman spectroscopy. In addition, calibration models between phenolic compounds and Raman spectroscopy were evaluated as rapid analytical methods. In this aspect, we have investigated a set of red and white wines from different Bulgarian regions (East, North, and South), which is to the best of our knowledge, the first study using Raman spectroscopy as a calibration or classification tool of wines.

## 2. Results and Discussion

**Figure 1:**
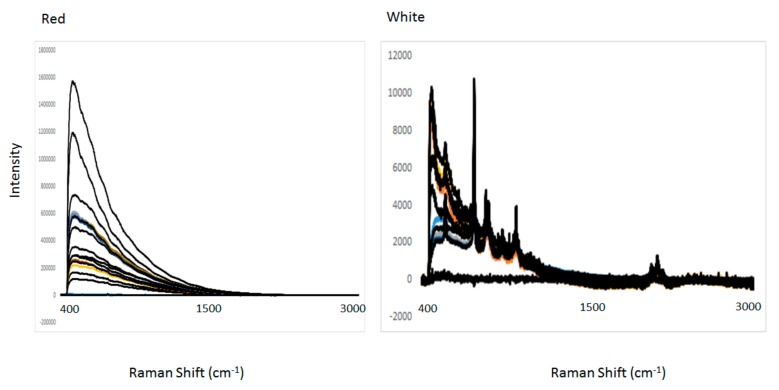
Typical spectra of red and white wine samples sourced from different varieties and regions and analyzed using Raman spectroscopy.

**Figure 2:**
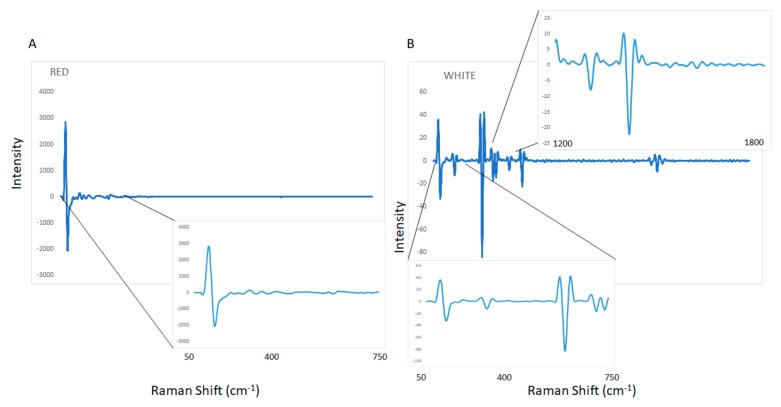
Second derivative applied to the Raman spectra of red (panel **A**) and white (panel **B**) wine samples.

Figure 1 shows the spectra of red and wine samples analyzed using Raman spectroscopy. The raw Raman spectra shows distinctive features associated with the intrinsic chemical compositional differences between the wine samples analyzed (e.g., phenolic compounds, anthocyanins, ethanol content). Figure 2 (panel A and B) shows the average of the second derivative of the Raman spectra of red and white wine samples, respectively. The main characteristic bands of the wine samples analyzed using Raman spectroscopy were associated with the main compounds present in the wine matrix such as ethanol (bands around 877, 1000, 1276, 1454, and between 2700 and 2980 cm^−1^) and water (stretching modes above 3200 cm^−1^ and bending mode at 1636 cm^−1^) [18,19,20,21,22]. Overall, the main spectral variability associated with the different wine styles and varieties analyzed can be noticeably observed in the Stokes (50–850 cm^−1^, 1600–1750 cm^−1^) or in the anti-Stokes range [18,19,20,21,22]. In particular, these low intensive bands can be associated with the presence of phenolic compounds as reported by other authors [18,19,20,21,22]. The observed differences might also be attributed to the different wine constituents and properties associated with variety (e.g., red vs. white wine).

It has been reported that the strong fluorescence background associated with the absorption of hydroxycinnamic acids (phenolics) and related phenolic compounds determine a Raman shift depending on the excitation [18,19,20,21,22], where, the observed emission in the wines is attributed to the excited state proton transfer [23,24].

Raman scattering (with 532 nm excitation) was suggested as the method to analyze white wine samples by other authors [22]. It was indicated that C–H stretching vibrations between 2600 and 3100 cm^−1^ are important to estimate ethanol and sucrose concentrations in wine using Raman spectroscopy [18,19,20,21,22]. The Raman scattering intensity in this region might be associated with both C–H and O–H bonds like those observed in the mid-infrared (MIR) region of the electromagnetic spectrum [18,19,20,21,22]. In the Raman spectrum, intense peaks might be observed at approximately 840 cm^−1^, 1030 cm^−1^, 1050 cm^−1^, and 1440 cm^−1^ [18,19,20,21,22]. The intensity peak observed around 880 cm^−1^ is probably originated from the CC stretching of ethanol while bands around 1250 and 450 cm^−1^ can be associated with the HCC and OCC bending, respectively [18,19,20,21,22]. Other peaks of weak intensity can be observed in the spectrum from 1050 to 1450 cm^−1^ and are presumably originated from hydroxycinnamic acids, such as caffeic, ferulic, p-coumaric, among others present in wines. It has been shown by other authors that Raman scattering around 1000 and 1600 cm^−1^ might be associated with the presence of phenolic compounds in white wine samples [18,19,20,21,22].

**Figure 3:**
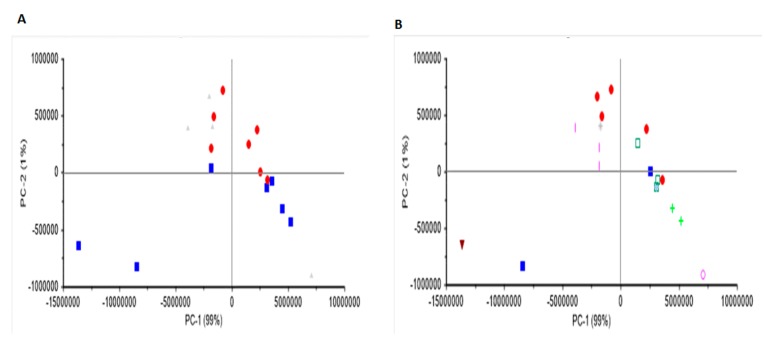
Principal component score plot of red wine samples analyzed using Raman spectroscopy. Panel A label by region (**Blue squares**: Danuban plains; **Red dots**: Upper Thracian plains; **Grey Triangles**: ungroup samples) and Panel B label by variety (**Blue squares**: Cabernet Franc; **Red dots**: Cabernet Sauvignon; **Inverted triangle**: Marselan; **Cross**: Melnik55; **Pink line**: Merlot; **Green cross**: Pinot Noir; **Green square**: Syrah).

**Figure 4:**
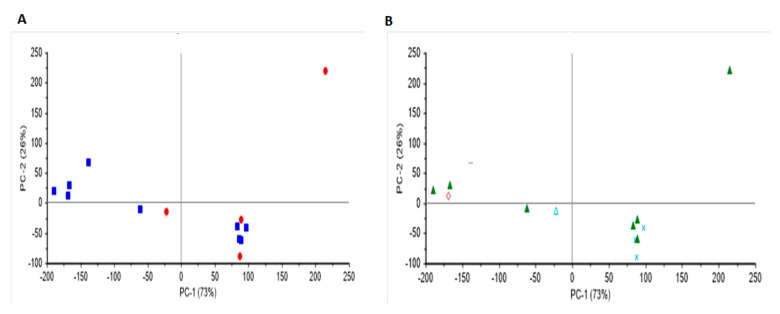
Principal component score plot of white wine samples analyzed using Raman spectroscopy. Panel A label by region (**Blue squares**: Danuban plains; **Red dots:** Upper Thracian plains) and Panel B label by variety (**Green triangle**: Chardonnay; **Line**: Muscat Ottonel; **Cross**: Sauvignon Blanc; **Blue triangle**: Tamjanka; **Diamond**: Viognier).

**Figure 5:**
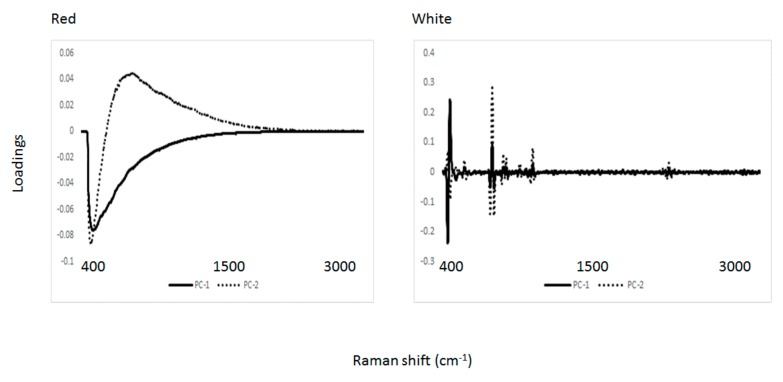
Loadings from the first two principal components derived from the principal component analysis of red and wine samples analyzed using Raman spectroscopy.

Principal component analysis was used to differentiate the wine samples according to region and variety. Figure 3 and Figure 4 show the score plot derived from the PCA analysis of both red and white wine samples scanned using Raman spectroscopy. For red wines, a separation between wines according to South and North Bulgarian regions was observed. This separation might be attributed to differences in the environmental conditions of the vineyard (e.g., soil chemical properties). Although Cabernet Sauvignon samples tend to cluster together, no clear separation-related variety was observed. It seems that the effect of variety can be distinguished by the background (fluorescence) band rather than Raman peaks (e.g., fluorescence from aromatic and phenolic compounds). Both principal component (PC)1 (99%) and PC2 (1%) contributed to explaining 100 percent of the variability in the PCA score plot. The separation between red wine varieties according to the region was observed along PC2. Overall, this data might suggest that the chemical and physical properties of the region/soil/environment from which the wine came from might have an influence on the Raman characteristics of the wine samples analyzed (e.g., wine varieties grown in different regions but with similar soil properties will display similar Raman characteristics). Unfortunately, no information about the chemical or physical properties of the soils was available. On the other hand, no clear separation related to either region or variety was observed for the set of white wine samples analyzed using Raman spectroscopy (PC1 73% and PC2 26%). It seems that Raman spectroscopy was not able to detect some of the compounds present in the white wine samples analyzed that contribute to the classification of samples according to either origin or variety. Similar results were reported by Magdas and collaborators [21]. Figure 5 shows the PCA loadings for the first two principal components for the red and white wine samples analyzed using Raman spectroscopy. The highest loadings observed in both PC1 and PC2 regions of the Raman spectrum are like those described in the above section. Overall, the loadings might be associated with the presence of phenolic compounds, ethanol, and water [18,19,20,21,22].

Table 1 shows the average, range, standard deviation, and coefficient of variation of the anthocyanins content, phenolic and flavonoid compounds measured in the set of red and white wine samples. A wide range in the concentration was observed as a consequence of the different varieties analyzed. These results agree with those reported by other authors [12,13,14,15,16,17]. Calibration models between phenolic compounds and Raman spectroscopy were developed using partial least squares (PLS) regression using the cross-validation (Table 2). Good calibration statistics were obtained for total phenolic (FC), total phenolic (Sommer), and phenolic acids were the coefficient of determination (R^2^) and the standard error in the cross-validation (SECV) were 0.81 (474.2 mg/dm^3^ gallic acid), 0.87 (526.6 mg/dm^3^ catechin equivalents), and 0.81 (44.8 mg/dm^3^ caffeic equivalents), respectively. The RPD values (> 2.5) indicated that the calibration models were adequate to measure phenolic compounds as low, medium, and high, qualitatively. Results from this study agree with those reported in Cabernet Sauvignon by other authors using Raman spectroscopy [23,24].

SECV: standard error of cross-validation, SD: standard deviation, RPD: residual predictive deviation (SD/SECV) value, R^2^: coefficient of determination in cross-validation. Overall, the performance of the calibration models developed clearly showed that phenolic compounds could be measured/predicted in wines using the combination of Raman spectroscopy and chemometrics. However, some other authors reported that Raman spectroscopy might be considered as a less sensitive technique comparing with other vibrational spectroscopic methods, although it might offers the advantage of being less sensitive to the presence of water in samples such as wine [18,19,20,21,22,23,24]. Overall, Raman spectroscopy might be considered as a good alternative to avoid the interference of these major components in the wine matrix to measure or predict different minor compounds in wine (e.g., phenolic compounds) that are of importance in determining provenance and the quality.

## 3. Materials and Methods

### 3.1. Wine Samples

Two sets of wine samples, namely a red set (n = 19) and a white set (n = 13) were sourced from different Bulgarian regions (North from the Danubian Plains, East from Black Sea, South from the Thracian Plains, and South-East from Struma Valley) (see Table 1), collected during the 2015 vintage and analyzed using both chemical (reference) and spectroscopy methods. The wines were obtained from both widespread and local cultivars. The red wine set contains Cabernet Sauvignon (n = 5), Merlot (n = 3), Syrah (n = 3), Pinot Noir (n = 2), Cabernet Franc (n = 2), and one sample of each Marselan, Egiodola, Melnik 55, and Shiroka Melnishka Loza. The collection of white wines contains Chardonnay (n = 7), Sauvignon Blanc (n = 3), and one sample of each Muscat Ottonel, Viognier, and Muscat Blanc à Petits Grains. The detailed geographic origin of the samples and their composition is shown in Table 1.

### 3.2. Chemical Analyses

The concentration of wine phenolics was estimated by analyzing total phenol content by the Folin-Ciocalteu procedure [25], as implemented in method OIV-MA-AS2-10 [26]. Total phenols, phenolic acids, and flavonoids were determined by spectrophotometric measurement at 280 nm, according to Sommers [27]. Anthocyanins and catechins were evaluated according to the method reported by Ribérau–Gayon and Stonestreet [28] and Pompei and Peri [29], respectively. The alcohol content, pH value, and acidity have been determined according to the methods OIV-MA-AS312-01B, OIV-MA-F1-06, and OIV-MA-AS313-01 of The International Organization of Vine and Wine [26], respectively. All used chemicals and solvents were of analytical grade.

### 3.3. Raman Spectroscopy

The Raman spectra of the wine samples were collected using an Avantes AVA-RAMAN-785TEC portable Raman spectrometer (Avantes, Oude Apeldoornseweg 28. NL-7333 NS, Apeldoorn, The Netherlands) using a 1 cm quartz cell (Apeldoorn, The Netherlands). The instrument has a solid-state 500 mW laser 785 nm, full-width half-maximum of 0.2 nm. An AvaRaman-PRB-785 focusing probe has been used for the measurements. The instrument was controlled using the AvaSoft-Raman stand-alone software for the AvaRaman system. The spectra were recorded at 20 s integration time and averaged after 20 scans.

### 3.4. Statistical and Multivariate Data Analysis

The Raman spectra were exported from the Avantes software in csv format to The Unscrambler software (Version X, CAMO ASA, Oslo, Norway) for chemometric analysis. Principal component analysis (PCA) was performed to examine the dominant patterns in the Raman spectra of the wine samples analyzed. Calibration models between phenolic composition and Raman spectra were developed using the partial least squares (PLS) regression with full cross-validation [30,31]. The Raman spectra were transformed using the second derivative (Savitzky–Golay transformation, 10 points smoothing, and second-order filtering) before calibration models were developed. Calibration models were evaluated using the standard error of cross-validation, bias, slope, and residual predictive deviation (RPD) values (standard deviation/standard error of cross validation) [30,31].

## 4. Conclusions

As reported and discussed by other authors, Raman spectroscopy is still underexplored in the wine industry when compared with the other available vibrational spectroscopy techniques (e.g., NIR, MIR). However, in this study, we have demonstrated that this technique can be suitable for measuring phenolic compounds in both red and white wine samples. In addition, Raman spectroscopy was useful to distinguish between Bulgarian wine regions in the set of the red wine samples analyzed.

## Figures and Tables

**Table 1:** Anthocyanin content, total and flavonoid compounds measured in the set of red and white wine samples.

**Wine Sample**		Total Phenols* **mg/dm^3^** **Gallic Acid**			Total Phenols** **mg/dm^3^** **Catechin Equivalents**			Phenolic Acids** **mg/dm^3^** **Caffeic Equivalents**			**Index of Total Phenols a.u.**
		**Avg**	**SD**	**CV**	**Avg**	**SD**	**CV**	**Avg**	**SD**	**CV**	**Avg**
**Red Wines**											
1	Pinot Noir^3^	2110.3	32.0	1.5	2614.1	7.9	0.3	217.1	0.0	0.0	44.6
2	Pinot Noir^3^	2325.8	7.3	0.3	3262.5	17.8	0.5	239.8	0.5	0.2	51
3	Merlot^3^	2352.8	7.3	0.3	3324.4	13.6	0.4	258.5	0.5	0.2	51.2
4	Cabernet Sauvignon^3^	1796.0	26.4	1.5	2803.5	5.2	0.2	217.7	0.5	0.2	41.6
5	Cabernet Franc^3^	1957.6	7.3	0.4	2661.4	28.4	1.1	238.1	0.5	0.2	52.5
6	Syrah^3^	1787.0	26.4	1.5	2934.6	5.2	0.2	271.5	0.0	0.0	41.7
7	Marselan^3^	1876.8	19.4	1.0	2967.4	7.9	0.3	302.6	0.0	0.0	44.3
8	Egiodola^3^	2505.4	33.6	1.3	3564.9	18.6	0.5	259.0	0.9	0.4	55.1
9	Merlot^4^	2029.5	7.3	0.4	3022.1	10.7	0.4	242.0	0.5	0.2	47.2
10	Cabernet Sauvignon^4^	2747.9	48.1	1.7	3910.9	8.9	0.2	290.7	0.0	0.0	57.5
11	Cabernet Sauvignon^5^	2685.0	7.3	0.3	3677.8	3.0	0.1	266.4	0.0	0.0	55.3
12	Syrah^5^	1679.3	14.7	0.9	2625.0	7.9	0.3	243.7	0.5	0.2	41.6
13	Cabernet Franc^6^	2469.5	57.3	2.3	3655.9	18.6	0.5	327.5	1.7	0.5	55.1
14	Syrah^6^	2568.3	12.7	0.5	3994.7	13.6	0.3	330.9	0.9	0.3	62.2
15	Cabernet Sauvignon^7^	2720.9	7.3	0.3	3346.3	7.9	0.2	215.4	0.5	0.2	53.6
16	Cabernet Sauvignon^10^	2972.4	0.0	0.0	4111.3	22.5	0.5	273.7	0.9	0.3	62.8
17	Merlot^10^	2334.8	60.0	2.6	3317.1	7.9	0.2	247.7	0.9	0.4	49.1
18	Melnik 55^11^	3080.1	14.7	0.5	4369.9	5.9	0.1	250.5	0.5	0.2	63.5
19	Shiroka Melnishka Loza^12^	1849.9	58.2	3.1	584.9	3.0	0.5	114.1	0.5	0.4	11.1
**White Wines**											
20	Sauvignon Blanc^1^	269.4	5.7	2.1	406.4	10.7	2.6	53.0	0.9	1.7	7.5
21	Chardonnay^1^	318.8	1.3	0.4	384.6	3.0	0.8	57.5	0.5	0.8	9
22	Muscat Ottonel^2^	251.4	6.3	2.5	206.1	7.9	3.8	33.7	0.5	1.4	6.5
23	Sauvignon Blanc^3^	273.9	0.7	0.3	333.6	10.7	3.2	55.8	0.8	1.4	7.8
24	Chardonnay^3^	366.4	3.2	0.9	435.6	10.7	2.5	76.2	0.9	1.2	9.6
25	Chardonnay^3^	332.3	0.7	0.2	380.9	10.7	2.8	64.3	0.5	0.7	9.3
26	Chardonnay^3^	368.2	3.2	0.9	486.6	7.9	1.6	79.0	0.0	0.0	10.5
27	Chardonnay^3^	371.8	2.9	0.8	446.5	3.0	0.7	79.0	0.5	0.6	10
28	Viognier^3^	337.6	3.8	1.1	351.8	13.6	3.9	50.7	0.9	1.8	8.2
29	Muscat Blanc à Petits Grains^5^	336.8	2.9	0.9	548.5	7.9	1.4	105.0	0.5	0.4	10.8
30	Chardonnay^5^	229.9	0.7	0.3	355.4	13.0	3.6	47.3	1.2	2.6	8
31	Sauvignon Blanc^6^	238.0	5.7	2.4	282.6	3.0	1.1	54.1	0.5	0.9	8.2
32	Chardonnay^6^	327.8	1.3	0.4	453.8	5.2	1.1	46.2	0.5	1.0	9.1
	**Wine Sample**		**Flavonoid** **mg/dm^3^** **Catechin Equivalents**			**Anthocyanins** **mg/dm^3^** **Anthocyanins**			**Catechins** **mg/dm^3^** **(±) Catechin**		
			**Avg**	**SD**	**CV**	**Avg**	**SD**	**CV**	**Avg**	**SD**	**CV**
	**Red Wines**										
1	Pinot Noir^3^		1682.6	7.9	0.5	232.4	1.6	0.7	930.4	26.9	2.9
2	Pinot Noir^3^		2233.9	15.9	0.7	197.5	0.8	0.4	1443.1	31.0	2.1
3	Merlot^3^		2215.6	11.7	0.5	240.6	1.1	0.5	1329.1	41.0	3.1
4	Cabernet Sauvignon^3^		1869.6	5.5	0.3	298.0	0.5	0.2	1025.3	55.9	5.5
5	Cabernet Franc^3^		1640.1	26.4	1.6	191.7	1.1	0.6	1101.3	41.0	3.7
6	Syrah^3^		1770.0	5.2	0.3	365.5	0.8	0.2	759.5	31.0	4.1
7	Marselan^3^		1669.2	7.9	0.5	274.7	1.0	0.3	512.7	15.5	3.0
8	Egiodola^3^		2453.6	14.8	0.6	303.0	1.4	0.5	987.4	41.0	4.2
9	Merlot^4^		1983.7	8.8	0.4	213.4	0.5	0.3	835.5	15.5	1.9
10	Cabernet Sauvignon^4^		2663.7	8.9	0.3	287.5	0.5	0.2	1424.1	41.0	2.9
11	Cabernet Sauvignon^5^		2535.0	3.0	0.1	346.5	1.9	0.5	1595.0	53.7	3.4
12	Syrah^5^		1579.3	6.0	0.4	328.6	0.6	0.2	987.4	26.9	2.7
13	Cabernet Franc^6^		2250.9	12.9	0.6	386.8	1.1	0.3	1746.9	55.9	3.2
14	Syrah^6^		2575.1	9.8	0.4	466.8	1.0	0.2	2031.7	41.0	2.0
15	Cabernet Sauvignon^7^		2422.1	9.5	0.4	284.4	1.4	0.5	2221.5	31.0	1.4
16	Cabernet Sauvignon^10^		2936.9	18.5	0.6	281.7	1.1	0.4	2316.5	53.7	2.3
17	Merlot^10^		2254.5	9.5	0.4	196.7	1.7	0.9	1879.8	41.0	2.2
18	Melnik 55^11^		3295.2	5.2	0.2	292.2	1.1	0.4	1310.1	55.9	4.3
19	Shiroka Melnishka Loza^12^		95.5	4.3	4.5	6.6	0.5	8.3	1898.8	41.0	2.2
	**White Wines**										
20	Sauvignon Blanc^1^		179.2	6.9	3.9				48.6	1.6	3.4
21	Chardonnay^1^		138.0	4.3	3.1				54.9	1.3	2.3
22	Muscat Ottonel^2^		61.5	6.0	9.8				57.7	2.7	4.7
23	Sauvignon Blanc^3^		94.2	7.5	7.9				51.6	2.2	4.3
24	Chardonnay^3^		108.8	6.9	6.4				57.7	1.1	1.9
25	Chardonnay^3^		105.2	8.8	8.4				55.4	2.7	4.9
26	Chardonnay^3^		147.7	7.9	5.3				67.6	1.1	1.6
27	Chardonnay^3^		107.6	4.3	4.0				53.2	1.6	3.1
28	Viognier^3^		134.3	9.8	7.3				62.3	1.6	2.6
29	Muscat Blanc à Petits Grains^5^		97.9	6.0	6.2				38.7	1.9	4.8
30	Chardonnay^5^		152.5	9.8	6.4				42.5	2.2	5.3
31	Sauvignon Blanc^6^		50.5	1.0	2.0				42.5	1.6	3.9
32	Chardonnay^6^		255.7	3.6	1.4				33.4	1.2	3.7

* by Folin–Ciocalteu procedure; ** by Sommers procedure; Superscript refers to collecting sites: 1-Suvorovo (Varna Province); 2-Pirgovo (Rouse Province); 3-Orjahovo (Vratsa Province); 4-Levunovo (Blagoevgrad Province); 5-Topoli dol (Pazardzhik Province); 6-Brestnik (Plovdiv Province); 7-Starosel (Plovdiv Province); 10-Tsernodab (Haskovo Province); 11-Gen. Todorov (Blagoevgrad Province); 12-Vranja (Blagoevgrad Province). According to the Bulgarian Wine and Spirit Drinks Act (2014, https://www.mi.government.bg/library/index/download/lang/en/fileId/83), the collecting sites belong to the wine-growing zone North “Danubian Plains” (2, 3), East “Black Sea” (1), South “Thracian Plains” (5, 6, 7, 10), and South-East “Struma Valley” (4, 11, 12).

**Table 2:** Calibration statistics for the prediction of total phenolics in the set of wine samples analyzed using Raman spectroscopy.

	R^2^	SECV	SD	Slope	Bias	RPD
Total Phenolic (FC) mg/dm^3^ Gallic Acid	0.81	474.2	1048	0.82	2.27	2.2
Total Phenolic (Sommer) mg/dm^3^ Catechin Equivalents	0.87	526.6	1534	0.91	19.4	2.9
Phenolic Acid mg/dm^3^ Caffeic Equivalents	0.81	44.8	102	0.84	0.81	2.3
